# Vascular smooth muscle cell proliferation depends on caveolin-1-regulated polyamine uptake

**DOI:** 10.1042/BSR20140140

**Published:** 2014-11-21

**Authors:** Mario Grossi, Catarina Rippe, Ramasri Sathanoori, Karl Swärd, Amalia Forte, David Erlinge, Lo Persson, Per Hellstrand, Bengt-Olof Nilsson

**Affiliations:** *Department of Experimental Medical Science, Lund University, Lund, Sweden; †Department of Cardiology, Clinical Sciences, Lund University, Lund, Sweden; ‡Department of Experimental Medicine, Second University of Naples, Naples, Italy

**Keywords:** caveolin-1, cell cycle, ornithine decarboxylase, polyamine transporter, polyamine, vascular smooth muscle cell, ASMC, aortic smooth muscle cell, Cav-1, caveolin-1, CEA, carotid endarterectomy, DFMO, difluoromethylornithine, DMEM, Dulbecco’s modified Eagle’s medium, HBSS, Hanks balanced salt solution, [^3^H]Put, [^3^H]putrescine, HRP, horseradish peroxidise, [^3^H]Spd, [^3^H]spermidine, HSP90, heat-shock protein 90, KO, knockout, ODC, ornithine decarboxylase, PI, propidium iodide, qRT-PCR, quantitative real-time PCR, VSMC, vascular smooth muscle cell, WT, wild-type

## Abstract

Much evidence highlights the importance of polyamines for VSMC (vascular smooth muscle cell) proliferation and migration. Cav-1 (caveolin-1) was recently reported to regulate polyamine uptake in intestinal epithelial cells. The aim of the present study was to assess the importance of Cav-1 for VSMC polyamine uptake and its impact on cell proliferation and migration. Cav-1 KO (knockout) mouse aortic cells showed increased polyamine uptake and elevated proliferation and migration compared with WT (wild-type) cells. Both Cav-1 KO and WT cells expressed the smooth muscle differentiation markers SM22 and calponin. Cell-cycle phase distribution analysis revealed a higher proportion of Cav-1 KO than WT cells in the S phase. Cav-1 KO cells were hyper-proliferative in the presence but not in the absence of extracellular polyamines, and, moreover, supplementation with exogenous polyamines promoted proliferation in Cav-1 KO but not in WT cells. Expression of the solute carrier transporters Slc7a1 and Slc43a1 was higher in Cav-1 KO than in WT cells. ODC (ornithine decarboxylase) protein and mRNA expression as well as ODC activity were similar in Cav-1 KO and WT cells showing unaltered synthesis of polyamines in Cav-1 KO cells. Cav-1 was reduced in migrating cells *in vitro* and in carotid lesions *in vivo*. Our data show that Cav-1 negatively regulates VSMC polyamine uptake and that the proliferative advantage of Cav-1 KO cells is critically dependent on polyamine uptake. We provide proof-of-principle for targeting Cav-1-regulated polyamine uptake as a strategy to fight unwanted VSMC proliferation as observed in restenosis.

## Abstract 2

We demonstrate that caveolin-1 negatively regulates vascular smooth muscle cell polyamine uptake and show that caveolin-1-regulated polyamine uptake is critical for proliferative advantage of caveolin-1 deficient cells, providing proof-of-principle for targeting this mechanism as a strategy to fight unwanted proliferation.

## INTRODUCTION

Restenosis of the carotid artery is common after CEA (carotid endarterectomy). It is reported that restenosis occurs in approximately 10% of patients within the first year following CEA, with even more patients developing restenosis in the longer term [1]. CEA is characterized by a complex interaction of proliferation and migration of smooth muscle cells and fibroblasts, extracellular matrix accumulation, and thrombus formation at the injured site, collectively causing narrowing of the blood vessel lumen. Since VSMC (vascular smooth muscle cell) proliferation is an important feature in the progression of restenosis, much effort is spent on finding ways to inhibit this process. In clinical practice, stents are coated with anti-cancer drugs such as the microtubule disruptor paclitaxel [2]. Owing to unwanted side-effects and therapy resistance there is a strong need for new and effective anti-proliferative drugs with low toxicity to combat restenosis.

There is mounting evidence for the involvement of polyamines (putrescine-Put, spermidine-Spd and spermine-Spn) in the regulation of VSMC proliferation and migration [3–6]. The current view is that delicately balanced biosynthesis, catabolism, uptake and excretion of polyamines are very important for health [7,8], and disturbance of this homoeostasis is associated with cancer and many other diseases [7,9]. In addition to a well-defined biosynthetic pathway, polyamines are internalized into cells by as yet incompletely defined mechanisms. Mammalian cells acquire polyamines in two ways: through *de novo* biosynthesis from basic amino acids and through the uptake of extracellular polyamines, a process that is mediated by polyamine transporters and permeases. Different classes of solute carrier transporters are implicated in polyamine uptake mechanisms [10]. Recently Uemura et al. [11] demonstrated that the solute carrier transporter Slc3a2 mediates polyamine uptake in intestinal epithelial cells through a Cav-1 (caveolin-1)-dependent mechanism [11]. It has also been reported that polyamine uptake is mediated by Cav-1-dependent endocytosis in colon cancer cells [12].

The Cav-1 protein is critical for caveolae, which are Ω- shaped cholesterol-rich signalling platforms within the cell membrane. Moreover, there is evidence for a dynamic role for Cav-1 in cell proliferation [13,14]. Disruption of the Cav-1 gene increases VSMC proliferation [15] and the increased proliferation of VSMC observed in human atheroma is associated with a decrease in Cav-1 expression [16]. This argues that Cav-1 plays a pivotal role in VSMC proliferation, suggesting that the loss of anti-proliferative control by Cav-1 may be important for restenosis. Knock-down of Cav-1 expression promotes uptake of polyamines in intestinal epithelial cells, indicating that Cav-1 is a negative regulator of polyamine uptake and that caveolae are platforms in the cell membrane for polyamine transport [11]. However, the physiological importance of the Cav-1-dependent polyamine uptake is unknown and has not been studied in VSMCs which have a high membrane density of caveolae. We showed recently that the local inhibition of ODC, a rate-limiting enzyme in the biosynthesis of polyamines, by α-DFMO (difluoromethylornithine) reduces vascular stenosis in a murine model of carotid injury, suggesting that DFMO can be used to prevent the unwanted proliferation of VSMCs in restenosis [17]. However, chronic treatment with DFMO may promote escape phenomena, including increased uptake of extracellular polyamines, providing necessary amounts of polyamines to the cells.

The present work aimed to clarify the role of Cav-1 in VSMC polyamine uptake and the physiological importance of this mechanism for cell proliferation and migration. We hypothesized that Cav-1 controls polyamine uptake and that VSMCs are critically dependent on this mechanism for their proliferative response. Our data demonstrate that Cav-1 negatively regulates VSMC polyamine uptake, and, moreover, we show that Cav-1-regulated polyamine uptake is critically important for the reported proliferative advantage of Cav-1 deficient cells.

## EXPERIMENTAL

### Animals

Cav-1 KO mice were originally obtained from the Jackson Laboratory (Bar Harbor, ME, U.S.A.) and were backcrossed on C57BL/6 [18]. Mice were maintained in homozygous breeding at the local animal facility at BMC, Lund, Sweden. WT C57BL/6 mice were purchased from Scanbur (Karlslunde) and matched for sex and age. Mice had free access to standard chow and water. Cav-1 KO and WT adult mice were euthanized with CO_2_ and blood was collected using cardiac puncture. Blood was allowed to clot for 30 min and serum was obtained by centrifugation (1500 ***g*** for 15 min). All experiments were approved by the local Animal Ethics Committee in Lund/Malmö (M433-12).

Adult Wistar rats, weighing 230–250 ***g*** were maintained in accordance with the guidelines of the NIH (Guide for the Care and Use of Laboratory Animals, 1976). All protocols were approved by the Animal Care and Use Committee of the Second University of Naples. Rats were acclimatized and quarantined for at least 1 week before undergoing surgery. They were anesthetized with intraperitoneal injection of 100 mg/kg ketamine and 0.25 mg/kg medetomidine and carefully placed onto a warm surface and positioned for surgery. All the surgical procedures were conducted with sterile techniques and vital signs were continuously monitored through a pulsioxymeter. Arteriotomy of rat common carotid artery was performed as already published [19].

### Cells and cell culture

ASMCs (aortic smooth muscle cells) were isolated from Cav-1 KO and control mice euthanized by CO_2_. Aortae were isolated and incubated for 30 min at 37°C in serum-free DMEM (Dulbecco's modified Eagle's medium) cell culture medium containing 1 mg/ml collagenase type 2 (Worthington Biochemical Corporation). The adventitia was then pulled off using forceps and the aorta was incubated for another 2 h in DMEM with 2 mg/ml collagenase type 2 and 0.2 mg/ml elastase (Sigma). The primary ASMCs were cultured in the DMEM medium with addition of antibiotics (50 units/ml penicillin and 50 μg/ml streptomycin) and 10% (v/v)FBS and trypsinized [0.25% (w/v) trypsin] upon reaching confluence. The cells were used in passages 2-6. They grew in a ‘hill and valley pattern’ [20] typical for ASMCs and were characterized by positive staining for the smooth muscle cell markers calponin and SM22. The VSMCs were kept in a water jacketed cell incubator at 5% (v/v) CO_2_ in air at 37°C. The cells were made quiescent by omitting FBS for 24 h before DNA synthesis and crystal violet assays were ran.

### Polyamine uptake assay and measurement of polyamine contents

Polyamine uptake was determined using [^3^H]Put ([^3^H]putrescine, 10 μM) and [^3^H]Spd ([^3^H]spermidine, 5 μM). The cells were pulse-labelled with radioactive polyamines for 30 min at 37°C. The incubation was terminated by placing the cell-dishes on ice followed by washing twice with PBS containing 10 mM putrescine or 10 mM spermidine. The cells were lysed in 0.5 M NaOH and radioactivity was measured in a liquid scintillation counter (Beckman). Radioactivity was expressed as d.p.m. and normalized to the total protein concentration in each sample. Protein concentration was determined using a Bio-Rad protein assay kit based on the Lowry method [21]. For polyamine analysis, cells were washed in PBS, harvested using 0.25% trypsin, and then centrifuged at 2880 ***g*** for 5 min at room temperature. The cell pellets were sonicated twice for 10 s in PBS, and aliquots were mixed with equal volumes of 0.4 M perchloric acid, incubated at 4°C for 30 min, and then centrifuged at 18000 ***g*** for 2 min at room temperature. Chromatographic separation and quantitative determination of the polyamines in the cell extracts were done with HPLC (Hewlett Packard 1100) with o-phthalaldehyde as the reagent [6]. Cellular polyamine contents were normalized to total protein concentration in each sample.

### Determination of DNA synthesis

DNA synthesis was determined by measuring the incorporation of radiolabelled methyl-[^3^H]-thymidine (PerkinElmer) into newly synthesized DNA. The isotope (1 μCi) was included for 1 h before cell harvest. Cells were washed twice with TCA (trichloroacetic acid), lysed in 0.5 M NaOH and the radioactivity measured by liquid scintillation counting.

### Cell density

Cell density was determined by nuclear staining with crystal violet. The cells were fixed in HBSS (Hanks balanced salt solution) containing 1% (v/v) glutaraldehyde for 30 min and then incubated with 0.1% crystal violet for 30 min at room temperature. Unbound dye was removed by gently rinsing the plates in deionized water. The plates were air dried and inspected under a microscope. To dissolve the crystal violet pigments in the cell nuclei 10% (v/v) acetic acid was included, and then plates were incubated on a shaker at a slow speed for at least 5 min. The absorbance at 595 nm was recorded for each well using a microplate reader (Multiskan GO, Thermo Scientific).

### Determination of cell-cycle phase distribution by flow cytometry

Cav-1 KO and WT ASMCs were stained with PI (propidium iodide) by standard staining protocols. Cells were then trypsinized, washed in PBS, and fixed in ice-cold 70% (v/v) ethanol at −20°C overnight. Subsequent to washes, they were stained with PI/RNase for 15 min at room temperature and assayed by FACS using Accuri C6 (BD Biosciences). A total of 10000–20000 events were collected in the gated single-cell population that excluded debris and aggregates.

### Scratch-induced wound-healing assay

Cell migration was assessed using an *in vitro* wound assay in six-well plates. The wound was made by scraping off the cell monolayer with a sterile pipette tip across the centre of the well. Another scratch in the form of a straight line perpendicular to the first one was performed to create a cross without cells. Floating cells were removed by extensive washing with the warm medium. The scratched regions were photographed under a phase-contrast microscope (Nikon TMS) equipped with a 40× objective and a digital camera at the time of the scratch (0 h) and 24 and 72 h after the scratch. The remaining cell-free area was measured using the ImageJ software (NIH), and cell migration was calculated as the percent wound closure relative to the cell-free area at 0 h.

### Transwell migration assay

Cells were plated at a density of 5×10^4^ cells/insert in the serum-free medium in the upper transwell chamber (polycarbonate filters of 8-μm porosity, Corning). 750 μl culture medium with 10% FBS was added in the lower chamber and then the cells were left for 12 h in the cell incubator. The cells were fixed in HBSS containing 1% (v/v) glutaraldehyde for 30 min and then they were incubated with 0.1% crystal violet for 30 min at room temperature. Unbound dye was removed by gently rinsing the plates in deionized water. The cells were removed from the upper chamber by a cotton swab. Cells attached to the bottom of the filter were photographed in phase contrast. Crystal violet staining was determined as described above.

### Determination of ODC activity

Aorta, lung and kidney tissues from Cav-1 KO and WT mice were homogenized in ice-cold 0.1 M Tris/HCl pH 7.5, containing 2.5 mM dithiothreitol and 0.1 mM EDTA. The tissue homogenates were centrifuged at 20000 ***g*** for 20 min at 4°C. Aliquots of the supernatants were used to determine the ODC activity by measuring the release of [^14^C]CO_2_ from ^14^C-labelled ornithine in the presence of pyridoxal 5-phosphate (0.5 mM) and ornithine (0.5 mM). Radioactivity was expressed as d.p.m. and normalized to mg tissue in each sample.

### qRT-PCR (quantitative real-time PCR) and RT-PCR

Total RNA from Cav-1 KO and WT cells was isolated using miRNeasy mini kit (Qiagen), including on-column DNAse I digestion according to the manufacturer's instructions. The relative expression of target genes was analysed by one step real-time qPCR (StepOnePlus qPCR cycler, Applied Biosystems) using QuantiFast SYBR Green RT-PCR Kit (Qiagen, 204156). The following QuantiTect primer assays (Qiagen) were used: Gapdh (Mm_Gapdh_3_SG, QT01658692), Slc3a2 (Mm_Slc3a2_1_SG, QT00109914), Slc7a1 (Mm_Slc7a_1__1_SG, QT00099799), Slc13a4 (Mm_Slc13a4_1_SG, QT00113764), Slc43a1 (Mm_Slc43a1_1_SG, QT00251832), Cav-1 (Mm_Cav1_1_SG, QT00252007), ODC-1 (Mm_ODC1_1_SG, QT00126889). For rats, total RNA was extracted from injured and uninjured carotids at 3 days after arteriotomy using the RNAeasy minikit (Qiagen) according to the manufacturer's instructions. RT-PCR experiments were performed as described by Forte et al. [22]. GeneBank sequences for rat mRNAs and the Primer Express software (Applied Biosystem) were used to design primer pairs for Cav-1.

### Western blotting

Cells grown in six-well plates were washed with ice-cold PBS and lysed on ice using SDS sample buffer [60 mM Tris/HCl, pH 6.8, 2% (w/v) SDS, 10% (v/v) glycerol] containing phosphatase and protease inhibitors (BioRad). After protein determination (Bio-Rad DC™ protein assay) bromophenol blue and β-mercaptoethanol were added to the samples at final concentrations of 0.005 and 5%, respectively. Approximately, 15 μg of protein were loaded on Bio-Rad TGX 4-15% Criterion gels. Proteins were then transferred to nitrocellulose membranes (0.45 μm) using the Turboblot system (Biorad). Membranes were blocked in casein-blocking buffer, washed in TBS-T [20 mM Tris/HCl, 0.5 M NaCl, 0.05% (v/v) Tween 20, pH 7.5], and incubated with primary antibodies; Cav-1 (D46G3, Cell Signaling), Calponin (ab46794, Abcam), HSP90 (heat-shock protein 90; #610418, BD Transduction Laboratories). Secondary rabbit HRP (horseradish peroxidase)-conjugated antibodies (#7074, #7076, Cell Signaling) were used. Bands were visualized using the West Femto chemiluminescence substrate (Pierce) and images were acquired using the Odyssey Fc Imager (LI- COR Biosciences). HSP90 was used as a reference protein and all bands were normalized to the expression of this protein.

### Immunochemistry

Vessels were fixed in 4% buffered formaldehyde, dehydrated and embedded in paraffin. Five micrometre cross-sections were dewaxed, rehydrated with descending concentrations of ethanol and rinsed in distilled water. Antigen retrieval was performed by trypsin. Cells were grown in eight-chamber slides. Cells were fixed in 4% (w/v) paraformaldehyde for 5 min at room temperature, washed carefully in PBS and then permeabilized with 0.2% (v/v) Triton X-100. Tissue sections and cells were stained with a monoclonal rabbit Cav-1 antibody (Cell Signaling, 1:400), polyclonal goat ODC (Santa Cruz, 1:50), polyclonal rabbit SM22 (Abcam, 1:200) and calponin (Abcam, 1:200). The immunoreactive signal was visualized using secondary antibodies conjugated with HRP (Cell Signaling) or Alexa-Fluor-488 (Life Technologies) or Alexa-Fluor-555 (Invitrogen) at 1:200 dilution. The nuclei were counterstained with DAPI (4′,6-diamidino-2-phenylindole; Invitrogen) or with haematoxylin (Histolab). Fluorescence and DAB (diaminobenzidine; Dako) were analysed using an Olympus DP72 microscope equipped with a digital camera. The Olympus CellSensDimension software was used for morphometric analysis.

### Statistical analysis

Summarized data are presented as means±S.E.M. For experiments using cultured ASMCs, each culture dish represents one biological replicate, i.e. one observation (*n*=1). The number of replicate cultures for each experiment is indicated in legends to figures and the table. Statistical significance was calculated by Student's *t*-test and one-way ANOVA followed by Bonferroni's multiple comparison test for *post hoc* analysis as appropriate (GraphPad Software, Inc., San Diego, CA, U.S.A.). *P* values <0.05 were considered significant.

## RESULTS

### Characteristics of arteries and isolated ASMCs from Cav-1 KO and WT mice

**Figure 1:**
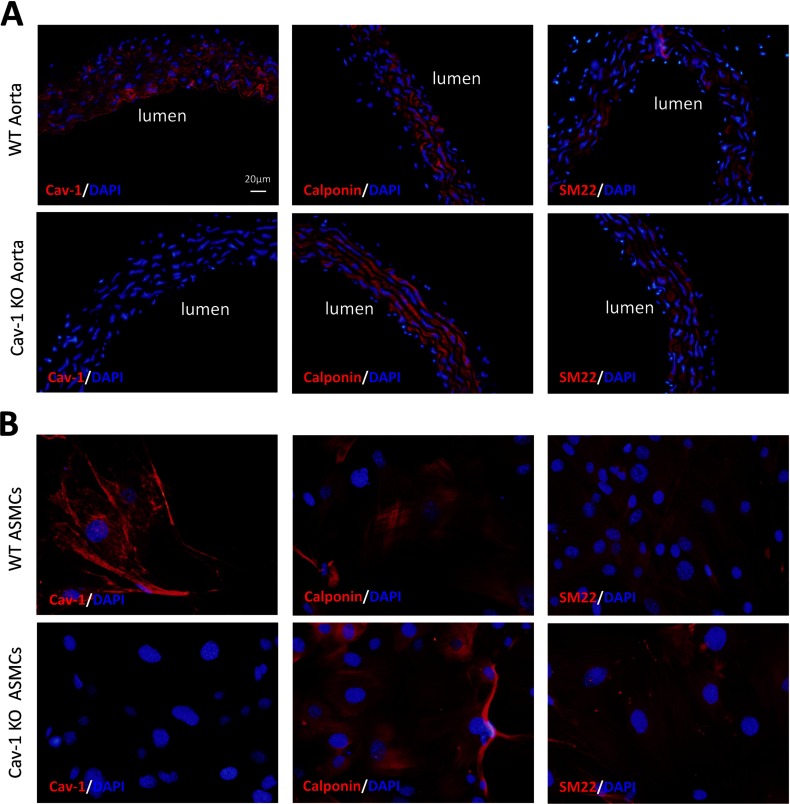
Characteristics of arteries and isolated ASMCs from Cav-1 KO and WT mice Both wild-type (WT) and caveolin-1 knockout (Cav-1 KO) mouse aorta (**A**) and aortic smooth muscle cells, ASMCs (**B**) express immunoreactivity for the contractile marker proteins calponin and SM22. No immunoreactive signal for Cav-1 is observed in Cav-1 KO aorta (**A**) and aortic smooth muscle cells (**B**). The nuclei were stained with DAPI. The scale bar in the upper left panel in figure A applies to all images.

As expected, ASMCs from Cav-1 KO mice showed no immunoreactivity for Cav-1, while both aorta and ASMCs from WT mice expressed Cav-1 (Figures 1A and 1B). The lack of Cav-1 in KO ASMCs was confirmed by Western blotting (Supplementary Figure S1). Both the Cav-1 KO and WT ASMCs showed characteristic ‘hill-and-valley’ growth pattern and the aorta and ASMCs from both Cav-1 KO and WT mice expressed the smooth muscle differentiation markers SM22 and calponin (Figures 1A and 1B).

### Cav-1 KO ASMCs show increased proliferation and migration rates compared with WT cells

**Figure 2:**
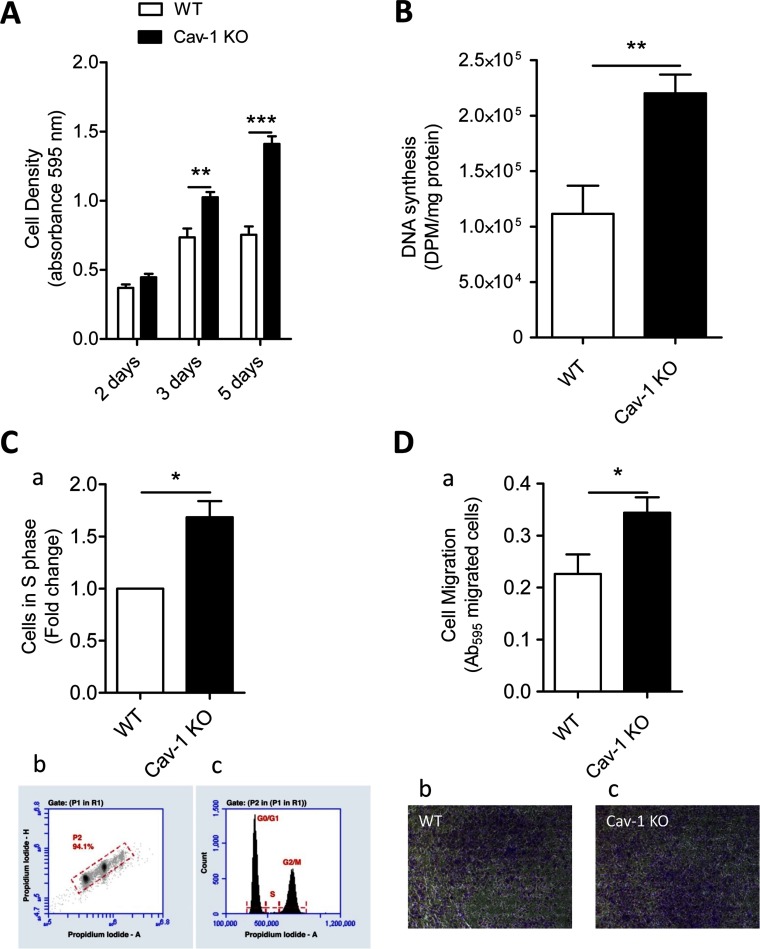
Cav-1 KO ASMCs show higher proliferation and migration rates than WT cells (**A**) Identical numbers of Cav-1 KO and WT cells were seeded at time 0 and cell density was assessed 2, 3 and 5 days after cell seeding. (**B**) DNA synthesis was assessed by measuring incorporation of ^3^H-thymidine into newly synthesized DNA 3 days after cell seeding. (**C**) The Cav-1 KO and WT cells were stained with propidium iodide (PI) by standard staining protocols 5 days after cell seeding. Flow cytometry of ASMCs derived from Cav-1 KO and WT mice show accumulation of Cav-1 KO cells in the S phase of the cell cycle (a). Dot plot of PI -Area/PI -Height with the gate set on the single cell population for DNA content analysis (Gate P2) for one representative sample of wild-type cells (b). This gate excludes debris and aggregates. Representative histogram of cells in G0/G1, S, and G2/M phases from WT cells (c). (**D**) Cell migration was evaluated by transwell chamber migration assay. Cav-1 KO cells migrate more than WT cells (a). Representative images of crystal violet staining of Cav-1 KO (c) and WT (b) cells. Three or four replicate cultures were performed. Values are presented as means±S.E.M. *n*=4–7 for each group. ** and *** represent *P*<0.01 and *P*<0.001, respectively.

Cav-1 KO and WT ASMCs were seeded in FBS-containing media, naturally containing a low concentration of polyamines (see below), to monitor cell proliferation. Cav-1 KO cell number increased with time after seeding, while the WT cells plateaued by 3 days (Figure 2A). The increase in cell density observed in WT cells between 2 and 3 days after cell seeding was associated with 30% down-regulation of Cav-1 protein expression (Supplementary Figure S1). The Cav-1 KO cell number was 30 and 60% higher than the WT cell number 3 and 5 days after seeding, respectively (Figure 2A). DNA synthesis was about 2-fold higher in Cav-1 KO compared with WT cells 3 days after cell seeding (Figure 2B). The cell-cycle phase distribution of Cav-1 KO and WT cells was analysed by flow cytometry of propidium iodide-stained nuclei 5 days after cell seeding (Figure 2C). Lack of Cav-1 increased the number of ASMCs in the S phase by about 1.5-fold (Figure 2C). Cav-1 KO cells showed about 1.5-fold higher migration rate compared with WT cells as assessed by the transwell migration assay (Figure 2D). Similar results were observed assessing cell migration in a scratch assay (results not shown). Taken together, these data show that Cav-1 plays an important but not critical role in both cell proliferation and migration.

### Exogenous putrescine promotes proliferation of Cav-1 KO but not WT ASMCs

**Figure 3:**
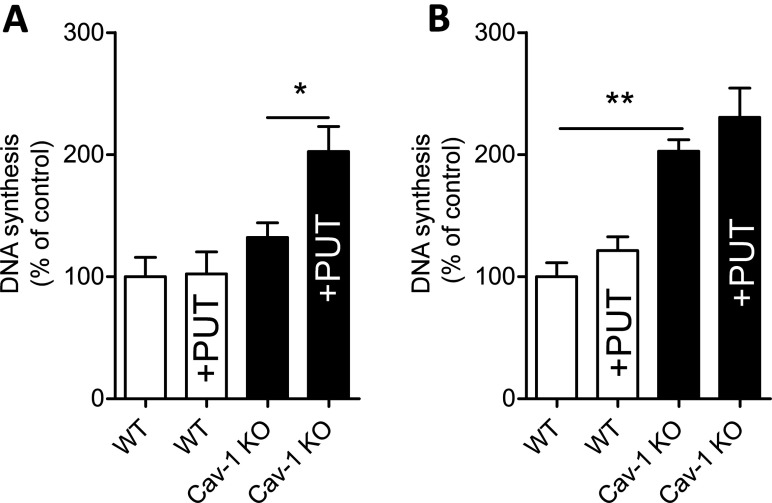
Cav-1 KO ASMCs cultured in the presence but not absence of polyamine show higher DNA synthesis rate than WT cells DNA synthesis was assessed by measuring incorporation of [^3^H]thymidine into newly synthesized DNA. The experiments were performed using 5% of dialysed FBS (**A**) lacking polyamines or non-dialysed 5% FBS containing polyamines, as demonstrated by HPLC (**B**). The molecular weight cut-off for the dialysed FBS was 6 kDa. Treatment with exogenous putrescine (Put, 50 μM) for 2 h enhances DNA synthesis in Cav-1 KO, but not WT cells cultured in dialysed FBS (**B**). Values are presented as the means±S.E.M. *n*=6–9 for each group. Three replicate cultures were performed.*and ** represent *P*<0.05 and *P*<0.01, respectively.

To demonstrate that exogenous polyamines play an important role in ASMC proliferation, cells were cultured in polyamine-free medium. Polyamine-free culture conditions were achieved by dialysing the FBS as described by Zeidan et al. [23]. Dialysed serum lacks small peptides and amines below 6 kDa [23]. Normal FBS contains polyamines, whereas no polyamines were detectable in the dialysed FBS as demonstrated by HPLC (results not shown). DMEM medium lacks polyamines; consequently, polyamine-free culture conditions were achieved through the combination of dialysed FBS with DMEM. No difference in DNA synthesis between Cav-1 KO and WT cells was observed when the cells were cultured in polyamine-free medium (Figure 3A). On the other hand, in the presence of added polyamines, the Cav-1 KO cells showed about 2-fold higher DNA synthesis rate compared with WT cells (Figure 3B). Supplementation with 50 μM Put for 2 h stimulated DNA synthesis in Cav-1 KO but not in WT cells cultured under polyamine-free conditions, whereas supplementation with Put had no effect on cellular DNA synthesis under normal culture conditions with polyamines present in the medium (Figures 3A and 3B). Taken together, these findings demonstrate that polyamine uptake plays a critical role for the proliferative advantage of Cav-1-deficient cells.

### ASMCs lacking functional Cav-1 gene show increased polyamine uptake

**Figure 4:**
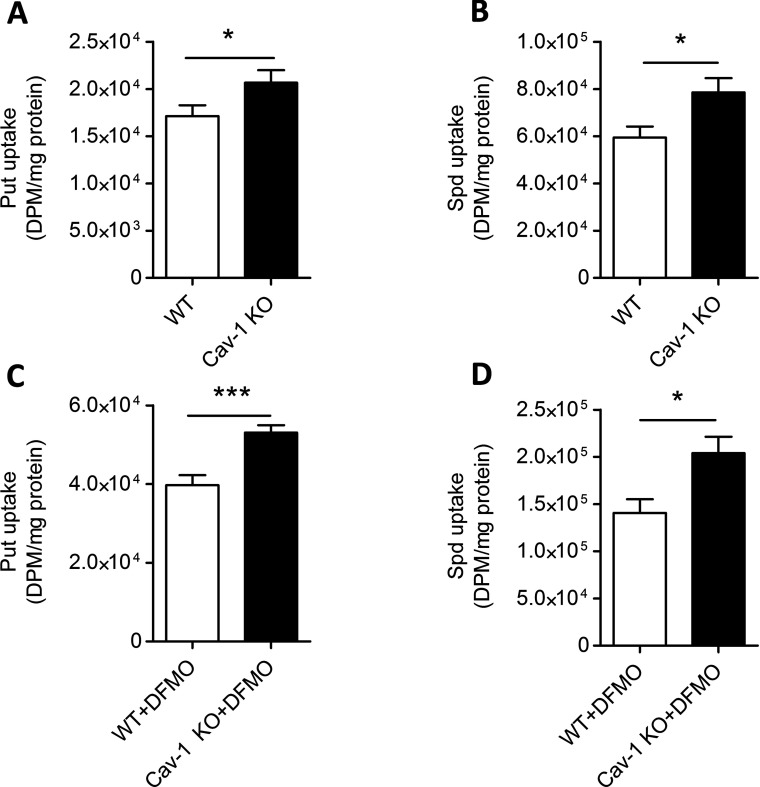
Polyamine uptake is higher in Cav-1 KO compared with WT cells Uptake of [^3^H]Put (10 μM) and [^3^H]Spd (5 μM) is higher in Cav-1 KO compared to WT ASMCs both without (**A**, **B**) and with DFMO (**C**, **D**). The cells were pulse-labelled with [^3^H]Put or [^3^H]Spd for 30 min after 2 days of treatment with or without 5 mM DFMO. Values are presented as the means±S.E.M. *n*=6–9 for each group. Three replicate cultures were performed.* and *** represent *P*<0.05 and *P*<0.001, respectively.

To directly test if the lack of Cav-1 expression affects the polyamine uptake in ASMCs, Cav-1 KO and WT cells were pulse-labelled with either [^3^H]Put or [^3^H]Spd after 2 days of treatment with or without 5 mM DFMO. Uptake of radio-labelled Put and Spd was about 20% higher in Cav-1 KO compared with WT cells (Figures 4A and 4B). It is well established that cellular polyamine depletion after DFMO treatment leads to the activation of polyamine uptake. Indeed, the treatment with DFMO for 2 days induced a 2–3-fold (*P*<0.001) increase of polyamine uptake in both Cav-1 KO and WT ASMCs (Figures 4C and 4D). However, even in the presence of DFMO, Put and Spd uptake was higher in Cav-1 KO than in WT cells (Figures 4C and 4D).

Cav-1 KO ASMCs showed higher intracellular endogenous polyamine levels compared with WT cells as demonstrated using HPLC (Table 1). Treatment with DFMO for 2 days dramatically attenuated cellular Put and Spd contents both in Cav-1 KO and WT cells (Table 1), showing that both Cav-1 KO and WT cells depend on endogenous synthesis of polyamines. In fact, DFMO reduced Spd more in Cav-1 KO than WT cells (Table 1). Moreover, when the cells were incubated for 2 h with exogenous Put, Cav-1 KO cells showed higher levels of intracellular Put compared with WT cells (WT: 4.56±0.07 nmol/mg protein versus KO:6.77±0.30 nmol/mg protein; *P*<0.01, *n*=3), arguing that cellular polyamine levels depend partly on polyamine uptake.

**Table 1:** Cav-1 KO ASMCs show higher intracellular polyamine levels compared with WT cells Treatment with DFMO for 2 days strongly attenuates cellular Put and Spd contents both in WT and Cav-1 KO cells. Values are presented as mean±S.E.M. of three observations in each group. ** and *** represent *P*<0.01 and *P*<0.001 for Cav-1 KO compared with WT cells, respectively. ^###^ represents *P*<0.001 for DFMO-treated compared with control cells.

	Putrescine (nmol/mg protein)		Spermidine (nmol/mg protein)		Spermine (nmol/mg protein)	
	Ctrl	DFMO	Ctrl	DFMO	Ctrl	DFMO
WT	0.69±0.02	0.00 ^###^	5.1±0.18	0.82±0.03^###^	2.50±0.14	2.53±0.43
Cav-1 KO	1.66±0.08***	0.00 ^###^	5.73±0.30	0.39±0.02***^###^	2.79±0.28**	3.57±0.27

### Cav-1 KO and WT mice show identical ODC expression and activity but different expression of polyamine transporter mRNAs

**Figure 5:**
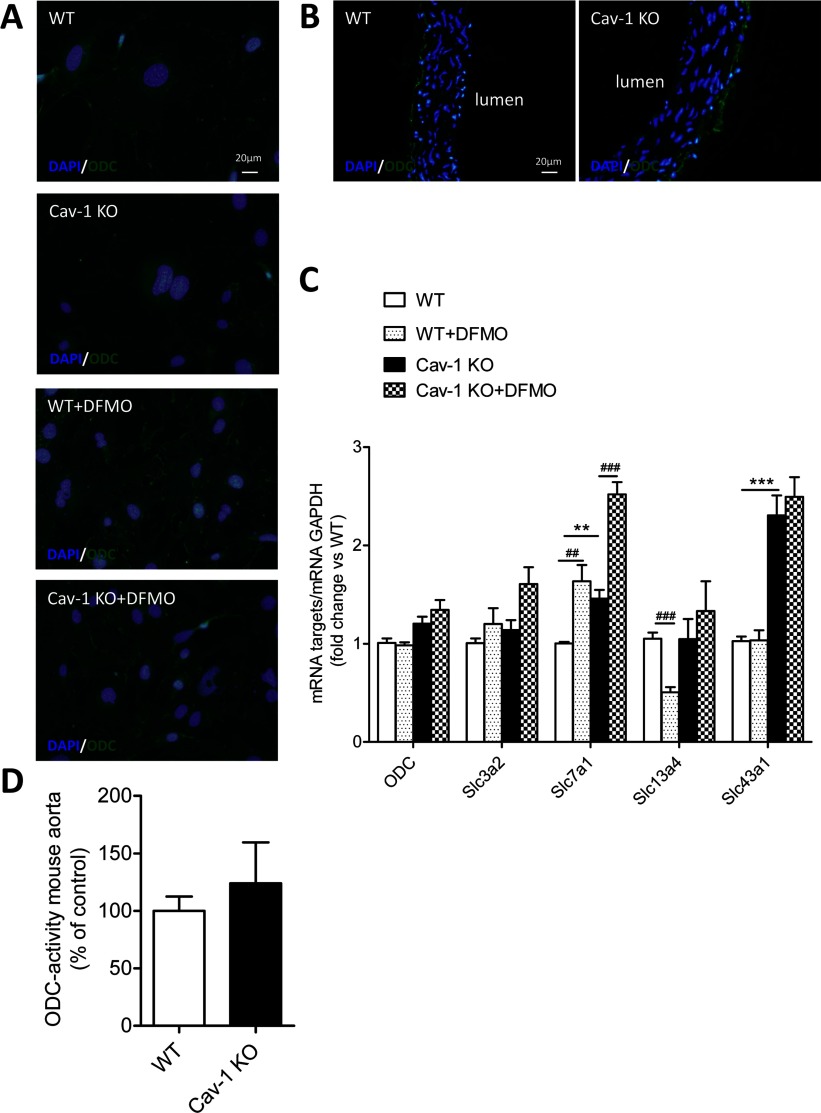
Cav-1 KO ASMCs show increased expression of Slc7a1 and Slc43a1 (**A**) Immunostaining for ODC shows that Cav-1 KO and WT ASMCs express a similar weak immunoreactive signal for ODC after 3 days of cell seeding. Both Cav-1 KO and WT cells treated for 2 days with 5 mM DFMO show a stronger signal than control cells not treated with DFMO. (**B**) Cav-1 KO and WT aorta show similar ODC immunoreactivity. (**C**) Quantitative PCR showing mRNA expression of ODC and the solute carrier transporters Slc3a2, Slc7a1, Slc13a4 and Slc43a1 in Cav-1 KO and WT cells treated with or without 5 mM DFMO for 2 days. Summarized data are presented as means±S.E.M., *n*=4 for each groups. Two replicate cultures were performed. ** and *** represent *P*<0.01 and *P*<0.001 for Cav-1 KO versus WT cells, respectively. ^##^ and ^###^ represent *P*<0.01 and *P*<0.001 for DFMO-treated cells versus their respective controls. (**D**) The ODC activity in mouse aorta, assessed by measuring the release of [^14^C]CO_2_ from [^14^C]-labelled ornithine was identical in Cav-1 KO and WT mice (*n*=6 for both groups). The scale bar in the upper left panel of figure A applies to all images.

ODC is the rate limiting enzyme in polyamine synthesis and it is inhibited by DFMO. Both Cav-1 KO and WT ASMCs showed a faint immunoreactivity signal for ODC (Figure 5A). Treatment with DFMO (5 mM) for 2 days increased the ODC signal for both Cav-1 KO and WT cells (Figure 5A). Immunoreactivity for ODC was detected in both Cav-1 KO and WT aorta with most intense staining near the adventitia (Figure 5B).

Next, we examined the ASMC expression of mRNAs coding for putative polyamine transporters belonging to four distinct classes of solute carrier transporters. Both Slc3a2 and Slc7a1 (also known as CAT-1) are implicated in polyamine uptake [10,11,24]. Slc13a4 and Slc43a1 were selected on the basis of their up-regulated expression in arrays on cavin-1-deficient lungs showing 80–90% reduction of Cav-1 (GEO accession number GSE43561) [25]. The Slc43a1 mRNA was up-regulated 2-3 times in Cav-1 KO compared with WT cells (Figure 5C). Slc7a1 mRNA level was about 50% higher in Cav-1 KO compared with WT cells (Figure 5C). Taken together, these data suggest that Slc7a1 and Slc43a1 are involved in Cav-1-dependent polyamine uptake. Interestingly, treatment with DFMO (5 mM) for 2 days increased Slc7a1, but not Slc43a1, expression in both Cav-1 KO and WT cells, suggesting that Slc7a1 mediates the DFMO-induced polyamine uptake (Figure 5C). On the other hand, DFMO reduced Slc13a4 expression in WT but not Cav-1 KO cells (Figure 5C). Incubation with 50 μM Put had the opposite effect on expression of Slc7a1 and Slc13a4 compared with DFMO (results not shown), indicating that these transporters indeed are sensitive to the intracellular polyamine levels.

ODC activity, assessed by measuring the release of [^14^C]CO_2_ from [^14^C]-labelled ornithine, was studied in aorta, lung and kidney tissues from Cav-1 KO and WT mice. Consistent with our immunofluorescence staining, ODC activity was identical in Cav-1 KO and WT aorta (Figure 5D). Enzyme activity was however about 50% lower in Cav-1 KO versus WT kidney, while ODC activity was similar in Cav-1 KO and WT lung tissue (Supplementary Figures S2A and S2B). Moreover, serum from both Cav-1 KO and WT mice contained polyamines (Supplementary Figure S2C).

### Cav-1 expression is dramatically reduced after injury

**Figure 6:**
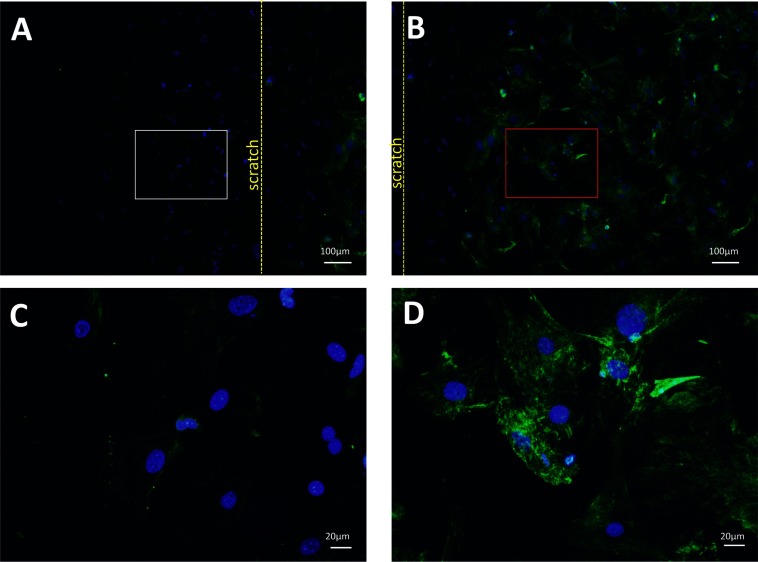
Cav-1 expression is dramatically reduced in proliferating ASMCs Immunocytochemical staining of Cav-1 in ASMCs obtained from WT mice using an *in vitro* wound assay (**A**–**D**). (**A**) proliferating cells (white rectangle) proximal to the border of the scratch (yellow dotted line). (**B**) non-proliferating cells (red rectangle) distal to the border of the scratch. The proliferating and non-proliferating cells are presented in (**C**) and (**D**), respectively, at big magnification. Very few Cav-1 positive cells are observed among cells proliferating out into the cell-free area (**A**, **C**), whereas confluent, non-proliferating, cells show high Cav-1 immunoreactivity (**B**, **D**).

**Figure 7:**
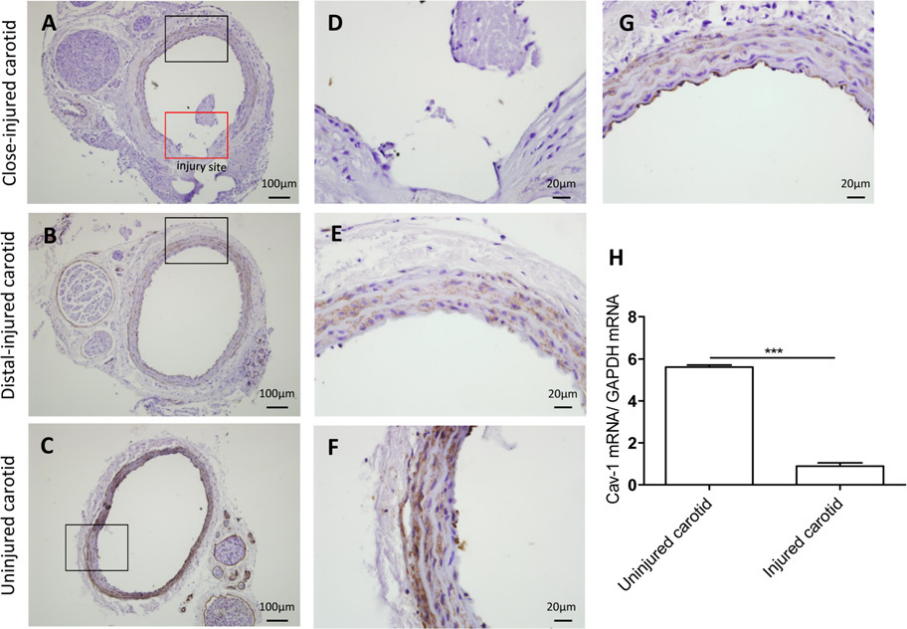
Cav-1 expression is dramatically reduced in injured rat carotids Representative immunohistochemical staining of Cav-1 in injured and uninjured rat carotids 3 days after arteriotomy. (**A**, **D**, **G)** carotid areas proximal to injury site represented by red and black rectangles in (**D**) and (**G**), respectively. (**B**, **E**) carotid areas distal to injury site. (**C**, **F**) Cross-sections from uninjured carotids. DAB-Cav-1-positive cells are stained in brown. Haematoxylin nuclei are counterstained in blue. (**H**) RT-PCR analysis of mRNA coding for Cav-1 3 days after carotid arteriotomy in carotids from injured and uninjured rats. Results are presented as means±S.E.M. *n*=4–5 animals.***P<0.001.

To investigate Cav-1 protein expression in proliferating and non-proliferating ASMCs, cells were submitted to an *in vitro* wound assay. Very few Cav-1 positive cells were observed among cells proliferating into the cell-free area (Figures 6A and 6C), while the confluent, non-proliferating cells in the same well showed high Cav-1 immunoreactivity (Figures 6B and 6D). The expression of Cav-1 protein (Figures 7A–7G) and mRNA (Figure 7H) were further investigated in a murine model of carotid arteriotomy [19]. Three days after the injury, RT-PCR and immunohistochemistry were performed on carotid samples at the arteriotomy site and on carotids from uninjured rats. Cav-1 expression showed a gradual decrease along the carotid cross-sections from the areas distal to the arteriotomy site (Figures 7B and 7E) to the areas proximal to the arteriotomy site (Figures 7A and 7G). Cav-1 was undetectable at the arteriotomy site (Figure 7D). No immunostaining was observed in any carotid area after omission of the primary Cav-1 antibody (results not shown). The down-regulation of Cav-1 protein expression in response to arteriotomy was confirmed at the mRNA level, as injured carotids showed a 6-fold decrease of mRNA coding for Cav-1 (*P*<0.001) compared with uninjured carotids (Figure 7H).

## DISCUSSION

The present work shows that VSMCs import polyamines from the extracellular space through a Cav-1-regulated mechanism. Cav-1 negatively regulates uptake of polyamines in primary ASMCs as reflected by higher total cellular polyamine levels, elevated uptake of radio-labelled polyamines and increased expression of carrier transporters in cells lacking Cav-1. Moreover, we show that the proliferative advantage conferred on cells by genetic ablation of Cav-1 is critically dependent on polyamine uptake. Our data are in agreement with previous work by Uemura et al. [11] who showed that Cav-1 negatively regulates polyamine uptake in intestinal epithelial cells, thus suggesting that this mechanism may be similar in several cell types expressing Cav-1. We show here that Cav-1-regulated polyamine uptake, at least in part, determines the proliferative response of VSMCs. Augmented proliferation of VSMCs in human atheroma has been associated with a decrease in Cav-1 expression [16]. The present work show that lack of Cav-1 expression promotes VSMC polyamine uptake and proliferation, suggesting that this may be a mechanism underlying the hyper-proliferation of human VSMCs observed in atheroma.

Disruption of the Cav-1 gene promotes polyamine uptake by VSMCs without affecting the endogenous vascular synthesis of polyamines as demonstrated by the unaltered ODC activity and ODC protein and mRNA expression in Cav-1 KO aorta and ASMCs. This is in line with previous reports showing that changes in ODC activity usually correspond to altered levels of enzyme protein [26]. Furthermore, we observed no difference in VSMC uptake of radiolabelled arginine between Cav-1 KO and WT cells (results not shown), providing evidence that Cav-1 specifically regulates the polyamine uptake. Indeed, we show that serum from Cav-1 KO as well as WT mice contains polyamines demonstrating that polyamines are available *in vivo* for cellular uptake.

The molecular mechanisms behind the uptake of polyamines are not completely understood [10,27]. Some candidate genes have been identified such as the CAT (cationic amino acid transporter) protein family and SLC (solute carrier) transporters. Here, we show that Cav-1 KO VSMCs express higher levels of Slc7a1 (CAT1) and Slc43a1 mRNAs compared with WT cells, suggesting that Slc7a1 and Slc43a1 mediate polyamine uptake in Cav-1 KO cells. These findings suggest that specific inhibitors of Slc7a1 and Slc43a1, unfortunately not yet available, might antagonize the hyper-proliferation observed in Cav-1 KO VSMCs. Furthermore, we show that DFMO-treatment, which reduces VSMC polyamine contents and stimulates polyamine uptake, up-regulates Slc7a1 mRNA both in Cav-1 KO and WT cells, suggesting that DFMO promotes uptake through this mechanism. Thus, Slc7a1 seems to be involved both in Cav-1-regulated and DFMO-mediated VSMC polyamine uptake. Importantly, DFMO enhanced Put and Spd uptake 2-3-fold in both WT and Cav-1 KO cells, suggesting that DFMO promotes polyamine uptake by VSMCs through a Cav-1-independent mechanism.

Interestingly, Cav-1 KO VSMC show elevated proliferation, DNA synthesis and cell migration although they clearly express the smooth muscle contractile marker proteins calponin and SM22, suggesting that Cav-1-dependent hyper-proliferation does not involve a transition from a contractile to a synthetic phenotype, as demonstrated in VSMCs stimulated to proliferate with e.g. FBS and PDGF [20,28]. This is supported by our earlier work showing elevated vascular contractile response to α_1_-adrenergic agonists but reduced depolarization-induced contraction in Cav-1 deficient mice, suggesting complex changes in contractile regulation in mice with low expression of Cav-1 [29,30]. The hyper-proliferation of Cav-1 KO cells was demonstrated by increased DNA synthesis, elevated cell density as well as increased proportion of cells in the S-phase of the cell cycle compared with WT cells. Both Cav-1 KO and WT cells increase in number between 2 and 3 days after seeding, but Cav-1 KO cells continue to increase in number over a longer time (5 days). This argues that proliferation is inhibited at day 5 post-seeding due to contact inhibition in WT but not Cav-1 KO cells. Moreover, our data demonstrate that the increase of cell density which is seen in WT cells between 2 and 3 days after cell seeding is associated with a decrease of Cav-1. In fact, fibroblasts (NIH 3T3) treated with Cav-1 antisense oligonucleotides show loss of contact inhibition [31]. Our results suggest that Cav-1 may also regulate contact inhibition in ASMCs.

In the present study, we further demonstrate decreased expression of Cav-1 in a murine model of carotid injury using both immunohistochemistry and RT-PCR. Previously, we showed that VSMCs are induced to proliferate after injury [17]. Reduced Cav-1 mRNA and protein expression and loss of caveolae are common features of cells transformed by oncogenes [32]. Accordingly, we observed that Cav-1 expression is inversely correlated with proliferation of vascular smooth muscle cells both *in vitro* and *in vivo*, suggesting that Cav-1-regulated polyamine uptake may be responsible for hyper-proliferation of vascular smooth muscle cells in vascular disease and restenosis. We identified two molecular targets, Slc43a1 and Slc7a1, which are up-regulated at mRNA level in Cav-1 KO compared to WT cells. Interestingly, the Slc7a1 showed early up-regulation after arteriotomy [33], suggesting that this mechanism is indeed important also *in vivo*.

In conclusion, Cav-1 is involved in the regulation polyamine uptake in VSMC. Our findings raise the intriguing possibility that silencing or inhibiting polyamine transport proteins or using Cav-1 gene delivery to injured vessels to prevent the Cav-1 decrease may represent a pharmacological approach for inhibition of proliferation of vascular smooth muscle cells in restenosis.

## Online data

Supplementary data
