# Supplementary figures and images for: Vascular smooth muscle cell proliferation depends on caveolin-1-regulated polyamine uptake
Source: Biosci Rep. 2014 Nov 21;34(6):e00153. doi: 10.1042/BSR20140140 (PMC4240025; doi:10.1042/BSR20140140)

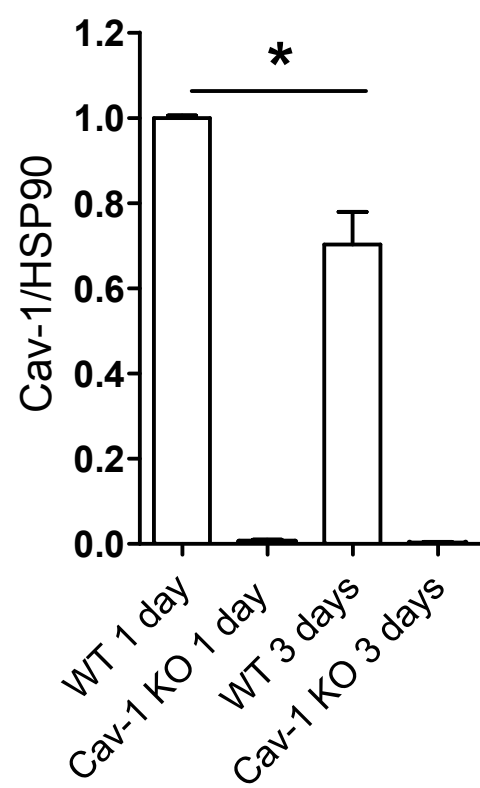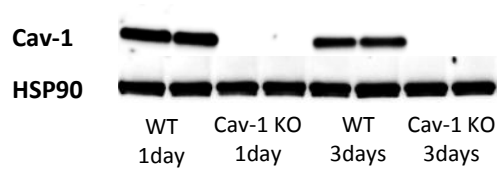

**A**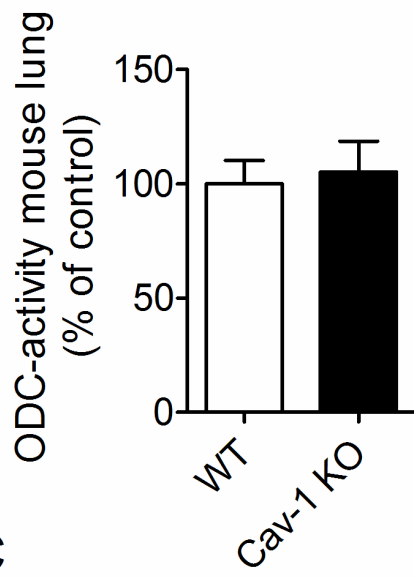**B**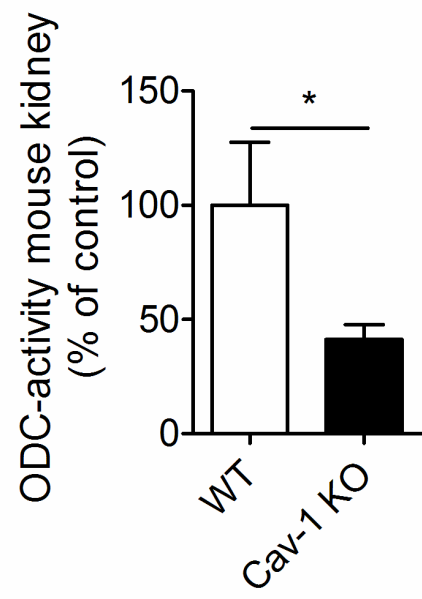**C**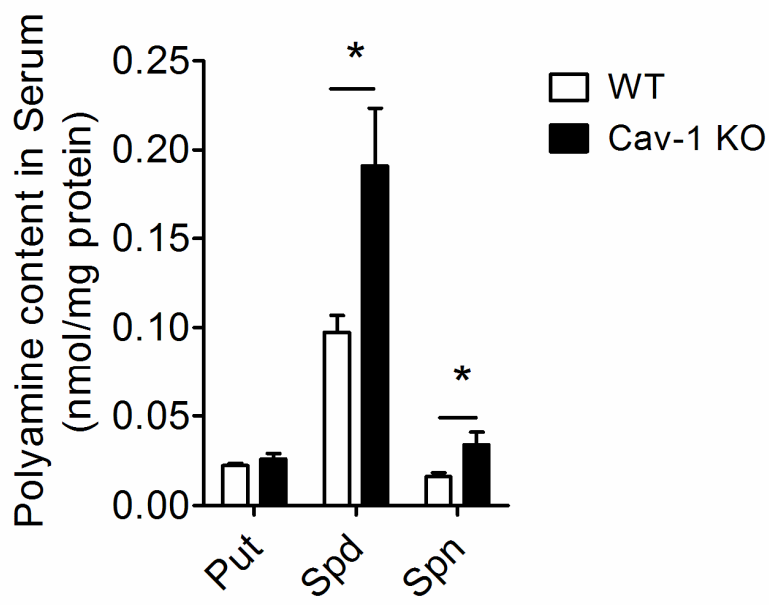

Supplement: Supplementary data [file bsr034e153ntsadd.pdf]
